# Mutanalyst, an online tool for assessing the mutational spectrum of epPCR libraries with poor sampling

**DOI:** 10.1186/s12859-016-0996-7

**Published:** 2016-04-04

**Authors:** Matteo Paolo Ferla

**Affiliations:** Formerly Department of Biochemistry, University of Otago, Dunedin, New Zealand; Present address: Biosyntia, DTU Centre for Biosustainability, Hørsholm, Denmark

**Keywords:** Enzyme engineering, Mutagenesis, Directed evolution, Mutational spectrum, Error-prone mutagenesis, Standard error, Confidence, Small sampling

## Abstract

**Background:**

Assessing library diversity is an important control step in a directed evolution experiment. To do this, a limited amount of colonies from a test library are sequenced and tested. In the case of an error-prone PCR library, the spectrum of the identified mutations — the proportions of mutations of a specific nucleobase to another— is calculated enabling the user to make more informed predictions on library diversity and coverage. However, the calculations of the mutational spectrum are severely affected by the limited sample sizes.

**Results:**

Here an online program, called Mutanalyst, is presented, which not only automates the calculations, but also estimates errors involved. Specifically, the errors are calculated thanks to the complementarity of DNA, which means that a mutation has a complementary mutation on the other sequence. Additionally, in the case of determining the mean number of mutations per sequence it does so by fitting to a Poisson distribution, which is more robust than calculating the average in light of the small sampling size.

**Conclusion:**

As a result of the added measures to keep into account of small sample size the user can better assess whether the library is satisfactory or whether error-prone PCR conditions should be adjusted. The program is available at www.mutanalyst.com.

## Background

In a directed evolution experiment library diversity is a key factor for its success. The diversity of a library is the amount of unique variants and, as many of which are represented multiple times, the library diversity distinct from the library size. The library needs to be sufficiently diverse to find improved variants (‘winners’), while not too rich in mutations to be swamped by non-functional protein. Consequently, to best identify winning variants in an error-prone PCR (epPCR) library, the mutations need to be at an optimal frequency (an average of 5 to 10 mutations/kb) [1] and not overly biased towards certain bases; preferential mutation of AT bases has been reported with manganese mutagenesis [2].

One way to ensure an effective diversity of a mutagenised plasmid pool is to assess the diversity in a small test library. Despite its cost of time and reagents [3], this step is strongly recommended as it avoids heavily investing in a potentially suboptimal library further downstream [1]. A test library entails plating an *E. coli* culture transformed with the pool of variant plasmids for growth under non-selective conditions. The colony abundance is assessed and about 10–20 clones are sequenced in order to calculate the overall mutation frequency, the error rate of the polymerase, the individual mutation frequencies and the biases associated with them [1]. When extensive sampling is done a highly accurate picture of the diversity of the library is seen, including the identification of a variety of sequence-specific mutation hotspots and coldspot [4], however a rough sampling is generally sufficient to estimate the main contributors of the mutational spectrum of the library. However, one major issue that arises is that the numbers may not be accurate.

In order to simplify and add statistical depth to these calculations, a new online calculator is presented here. The program, Mutanalyst (available at www.mutanalyst.com; portmanteau of mutation and analyst), uses the gene sequence and the list of mutations found to calculate the mutation frequency per sequence, the specific mutational frequencies (normalised by nucleotide distribution) and various bias indicators tabulated and graphed (vide infra for details). A novel feature of this tool is that it estimates the error associated with the values found. This implementation is driven by the need to determine the accuracy of the calculations in light of the limited sampling of the test library. This benefits the user greatly as it gives an indication of the reliability of each parameter, and thus a more informed perspective in determining the suitability of the library.

## Implementation

The site is written to be compatible with modern browsers and Internet Explorer 8 or above with JavaScript enabled (default setting in all browsers). The site is a series of three static HTML files powered by client-side JavaScript. This was done in order to allow transparency in the calculations involved (explained in the site) and to allow a user to use the site offline (available at github.com/matteoferla). The operations of the site are explained in detail in the Appendix.

The program has five parts with two different starting points. One starting point allows the user to input the wild type sequence and the list of mutations in the sampled sequences (written either as 239A > T, the standard notation, or as A239T, nucleotides written with the protein notation — further details can be found on the webpage), to generate the analysis of the number of mutations per sequence.

## Results and discussion

The analysis of the number of mutations per sequence by Mutanalyst is improved compared to standard protocols [1]. It shows not only the average number of mutations, but it also calculates the mean (λ) of a Poisson distribution fitted to the value. This distribution approximates the PCR-distribution of Sun [5], but does not require the knowledge of the PCR efficiency. The latter value is important in light of the inevitable error arising from the small sampling size, which can be ameliorated by imposing this valid assumption of the distribution of the values. The number of mutations per sequence is a key indicator of the diversity of the library and is also used by another program, Pedel, to determine library completeness in terms of nucleic acid mutation coverage [6–8]. Together with nucleobase-specific mutational rates, the mean number of mutations per sequence is used both in Pedel-AA [8] and the library diversity program by Volles and Lansbury [9] to determine the library completeness in terms of amino acids. Albeit unaffiliated, the Mutanalyst output can be linked directly to Pedel-AA, circumventing the need for the user to copy the parameters manually.

In the third section, the user can input the nucleobase-specific mutational rates, which is otherwise obtained from the list of mutations and the wild type sequence. The resultant normalised nucleobase-specific mutational rates are displayed as both a table and as a diagram to concisely show the mutational spectrum.

The final section contains the standard indicators of bias with the addition of an error estimate. The calculation of errors is accomplished thanks to the complementarity of DNA. Specifically, a mutation from one base to another on one strand is matched by a complementary mutation on the opposite strand (e.g. A → G and T → C). Consequently, these two separate values can be taken as replicates from which to derive the errors in the values. The propagation of errors was done parametrically using Eqs. 1 and 2 with the assumption that the covariance is zero in light of the independence of each mutational event (further detail is found on the webpage).

1 $$ Var\left(x+y\right)=Var(x)+Var(y) $$

2 $$ Var\left(\frac{x}{y}\right)\approx \frac{Var(x)}{\mu_y^2}+\frac{\mu_x^2 \cdot \kern0.5em Var(y)}{\mu_y^4} $$

The indicators are as follows:

- the sum of the four transition frequencies, i.e. purine (R) to purine, pyrimidine (Y) to pyrimidine,
- the sum of the eight transversion frequencies, i.e. purine to pyrimidine, pyrimidine to purine,
- the ratio of transitions over transversions,
- the frequency of a weak-binding nucleobase pair (W: A and T) mutating,
- the frequency of a strong-binding nucleobase pair (S: G and C) mutating,
- the frequency of weak-binding nucleobases mutating to strong ones,
- the frequency of strong-binding nucleobases mutating to weak ones and
- the ratio of the latter two frequencies.

In particular the two ratios are the most important parameters as they cover the two largest sources of bias. Even though, in terms of possible combinations, there are twice as many transversions as transitions, the steric difference between purines and pyrimidines means that transitions occur less frequently than transversions. Due to the differences in binding strengths between weak-binding and strong-binding nucleobases, some epPCR methods more readily mutate weak-binding nucleotides. As a consequence, these two ratios are the two most crucial indicators of bias. Thanks to the estimation of the error associated with them, these values can be compared with other libraries that may have been unsuccessful or successful, to those from the brochure provided with a commercial enzyme (e.g. Genemorph II) or to a reference cut-off that the user may have chosen.

## Conclusion

This easy-to-use online tool was designed to automate the calculations involved while simultaneously adding conservative error values. Specifically, it simplifies laborious checking of sequence for mutations, tallying of mutations, normalisation steps and fiddly calculations of commonly used biases indicators. This is done with the addition of informative graphs and with the addition of statistical rigour. The graphs include a Sankey diagram to show the directions of the mutation. The statistics that goes beyond current standards, includes a Poisson fit for the distribution of mutations per sequence in order to force the data to not be overly affected by jackpot samplings, the estimation and the propagation of errors by taking advantage of the fact that a mutation on one strand is as likely as a mutation on the complementary strand. The main aim was to automate and to add statistical confidence to the values in order to give a better representation of the values, which are strongly affected by the small sampling and may be otherwise misleading.

### Ethical approval

No ethical approval was required for this study.

### Availability and requirements

The program is available at http://www.mutanalyst.com. The source code can also be found at https://github.com/matteoferla/mutant_calculator.

## Appendix

### Statistical notes

The derivation of the formula for the variance of a ratio is explained in this walk-through.

The mean (μ) and variance (Var(x) or σ^2^) of a distribution (random variable) are variables that describe the function — technically they are the first and second moments respectively. The expected value (E(x)) is the average of a series of values sampled of a random variable (x). The mean is the expected value of the values themselves (Eq. 3), while the variance is the expected variable of the squared differences from the mean (Eq. 4).

3 $$ {\mu}_x={\displaystyle \sum_{i=1}^n}\frac{\left({x}_i\right)}{n}=E(x) $$

4 $$ Var(x)={\displaystyle \sum_{i=1}^n}\frac{{\left({x}_i-\mu \right)}^2}{n}=E\left[{\left(x-\mu \right)}^2\right] $$

To proceed we need to determine the variance of a function. The Eq. 4 can be easily rewritten as Eq. 5 (König–Huygens theorem) thanks to a binomial expansion and the rules of linearity of the expected value (Eqs. 6 and 7).

5 $$ Var(x)=E\left[{\left(x-\mu \right)}^2\right]=E\left({x}^2\right)-E{(x)}^2 $$

6 $$ \begin{array}{l}E(a)=a\ \mathrm{when}\ \mathrm{a}\ \mathrm{is}\ \mathrm{a}\ \mathrm{constant}\\ {}\kern4em \therefore \kern0.5em E\left(E(x)\right)=E(x)\end{array} $$

7 $$ E\left(x+y\right)=E(x)+E(y) $$

A function at a set point can be approximated to a polynomial, thanks to the Taylor series, which is an infinite sum, but for simplicity only the first two terms are shown in Eq. 8, which is what is known as a first-order approximation.

8 $$ f(x)\approx f\left(\mu \right)+f\hbox{'}\left(\mu \right)\left(x-\mu \right) $$

Rewritting Eq. 5 for a function and expanding each term as a Taylor series (Eq. 8), gives us Eq. 10. The first term of Eq. 10 is expanded by binomial expansion and then the expected value function is distributed to each member of the sum, whereas in the second term the expected values is distributed on two summands and then the square of the binomial is resolved. It should be noted that by definition the expected value of the (linear) difference from the mean is zero (Eq. 11). After that several terms cancel each other out giving the first-order approximation for the variance of a function (Eq. 12).

9 $$ Var\left(f(x)\right)=E\left(f{(x)}^2\right)-E{\left(f(x)\right)}^2 $$

10 $$ Var\left(f(x)\right)\approx E\left({\left[f\left(\mu \right)+f\hbox{'}\left(\mu \right)\left(x-\mu \right)\right]}^2\right)-E{\left(f\left(\mu \right)+{f}^{\hbox{'}}\left(\mu \right)\left(x-\mu \right)\right)}^2 $$

11 $$ E\left(x-\mu \right)=\mu - \mu =0 $$

12 $$ Var\left(f(x)\right)\approx {f}^{\hbox{'}}{(x)}^2\cdot Var(x) $$

Higher-order approximations can be done (e.g. second order, Eq. 13), but not only there is a diminishing return in precision, the complications increase dramatically.

13 $$ Var\left(f(x)\right)\approx {f}^{\hbox{'}}{(x)}^2\cdot Var(x)+f(x)\cdot f\hbox{'}\hbox{'}(x)\cdot Var(x) $$

Having proved the formula for the variance of a function (Eq. 12), we can proceed to determine the variance of a ratio. First, the ratio can be converted into logarithmic form (Eq. 14). The variance of a logarithm (Eq. 15) can be calculated from the approximation found (Eq. 12). This therefore allows the first order Taylor approximation of the variance of a ratio to be found.

14 $$ \ln \left(\frac{x}{y}\right)= \ln (x)- \ln (y) $$

15 $$ Var\left( \ln (x)\right)\approx {\left(\frac{1}{x}\right)}^2Var(x) $$

16 $$ Var\left(\frac{x}{y}\right)\approx \frac{Var(x)}{\mu_y^2}+\frac{Var(y)\cdot {\mu}_x^2}{\mu_y^4} $$

### Site map and description

This section details how the program works and its knowledge is not needed to use the program. It is intended as an overview in case a researcher wanted to alter it for a different purpose or copy a function from it.

Mutanalysis is composed of three Html pages:

- *Mutational_bias_calculator*
- *Mutation_counter*
- *Variance_notes*

Each page uses a central CSS file, mut.css, and two external style resources, *Font-Awesome* and the *Source Sans Pro* (*Google fonts*), two commonly used resources used in contemporary webpages.

The three html pages also use several external JavaScript (JS) resources:

- *JQuery*, an essential library that greatly simplifies JS coding.
- *Tooltip JS* (and *Tether JS* and *Drop JS*, and its style sheet), a library used to make tootips (notes on hover), which have several advantages over the inbuilt title attribute of html tags.
- *Google Charts*, a JS library that allows charts to be plotted, part of the Google Developers tool kit.
- *Google Analytics*, a JS widget that send asynchronously data to Google allowing the author to see what browsers are being used. At present, Mutanalyst is optimally viewed with Google Chrome on a Mac or Windows, but that may change in the future.

Additionally the page *Mutational_counter* uses a specific JS file, *mutationalCounter.js* while *Mutational_bias_calculator* uses two specific JS files:

- *mutationalBias.js*, a script that handles the calculations and does not interact with the document or utilise any other script.
- *mutationalAux.js*, a script that handles all the events of the buttons and other document interactions.

The key object in the *mutationalBias.js* calculations is called “mutball” (following after tarball etc.), which store all the variables and contains several keys that match the id of elements in the html document allowing *mutationalAux.js* to modify them without unnecessary coding. Its constructor is called mutagen. With a few exceptions (radio buttons, which do not call *mutationalBias.js*) it is recreated in case the user alters anything. The exception get the object via SessionStorage. The attributes can additionally be passed by URL query string. Some of the attributes are:

- *source*: a string noting whence the object was called.
- *sequence*: e.g. ATATCGG.
- *baseList*: e.g. G286A T306C A687T T880C\nWT\nWT.
- *freqMean*: mean frequency of number of mutations per sequence, a simple arithmetic average.
- *freqVar*: variance of number of mutations per sequence.
- *freqList*: array of the mutation counts (binned) of the rows of baselist.
- *freqΣ*: sum of number of mutations per sequence sampled.
- *freqλ*: Poisson distribution of number of mutations per sequence.
- *rawTable*: 4x4 nested arrays containing the mutation spectrum observed.
- *mutTable*: as above but normalised.
- *sumA*, sumT etc. the number of As in the sequence.
- *A2T* etc. number of incidents going from A to T. There are 16 of these. It is redundant with *rawTable*: but for html reasons it’s repeated.
- *size*: gene size in kb.
- *TsOverTv* and *TsOverTv_error*: transitions over transversions and its error. The keys with errors are as follows (they codes are: W = weak AT, S = strong GC, N = any Σ = sum)
  - o TsOverTv
  - o W2SOverS2W
  - o W2N
  - o S2N
  - o W2S
  - o S2W
  - o ΣTs
  - o Ts1
  - o Ts2
  - o ΣTv
  - o TvW
  - o TvN1
  - o TvS
  - o TvN2

The main methods are:

- *calcFreq(mutball)*: calculates the parameters associated with the number of mutations per sequence, in turn it calls various functions including fit(ordinate), which is a wrapper for the non-linear fitting fuction fminsearch (fun,Parm0,x,y,Opt), passing it the function of the Poisson distribution —i.e. if you want to change function tinker with fit(). fminsearch (fun,Parm0,x,y,Opt) is a small function adapted from JMat (GNU licence)
- *calcBias(mutball)*: calculates the mutational spectrum parameters.
- *Mutagen()*: returns a blank mutball object.

## Abbreviations

A, T, C, G, K, M, S, W, P, Y: IUPAC nucleobase abbreviations for adenine, thymine, cytosine, guanine, guanine + thymine, adenine + cytosine, guanine + cytosine, adenine + thymine, adenine + guanine, thymine + cytosine respectively
epPCR: error-prone polymerase chain reaction
